# Students Wearing Police Uniforms Exhibit Biased Attention toward Individuals Wearing Hoodies

**DOI:** 10.3389/fpsyg.2017.00062

**Published:** 2017-02-06

**Authors:** Ciro Civile, Sukhvinder S. Obhi

**Affiliations:** Social Brain, Body and Action Lab, Department of Psychology, Neuroscience and Behavior, McMaster UniversityHamilton, ON, Canada

**Keywords:** attention, police uniform, low-status, high-status, race effect

## Abstract

Police provide an essential public service and they often operate in difficult circumstances, requiring high-speed cognition. Recent incidents involving apparent profiling and aggressive behavior have led to accusations that the police are sometimes biased. Given that previous research has shown a link between clothing and cognition, we investigated the question of whether the police uniform itself might induce a bias in social attention. To address this question, and using a Canadian university student sample, we assessed whether wearing a police uniform biases attention toward black faces compared to white faces, and low-status individuals compared to high-status individuals. In Experiment 1 (*n* = 28), participants wore either a police-style uniform or mechanic overalls, and performed a shape categorization task in the presence of a distractor that could be either: a black face, a white face, a person wearing a hoodie (whom we propose will be associated with low SES), or a person wearing a suit (whom we propose will be associated with high SES). Participants wearing the police-style uniform exhibited biased attention, indexed by slower reaction times (RTs), in the presence of low-SES images. In Experiment 2 (*n* = 28), we confirmed this bias using a modified Dot-Probe task – an alternate measure of attentional bias in which we observed faster RTs to a dot probe that was spatially aligned with a low SES image. Experiment 3 (*n* = 56) demonstrated that attentional bias toward low-SES targets appears only when participants *wear* the police-style uniform, and not when they are simply exposed to it – by having it placed on the desk in front of them. Our results demonstrate that wearing a police-style uniform biases attention toward low-SES targets. Thus, wearing a police-style uniform may induce a kind of “status-profiling” in which individuals from low-status groups become salient and capture attention. We note that our results are limited to university students and that it will be important to extend them to members of the community and law-enforcement officers. We discuss how uniforms might exert their effects on cognition by virtue of the power and cultural associations they evoke in the wearer.

## Introduction

In the western world, police forces are generally respected and acknowledged for the crucial role they play in law enforcement and maintaining safe and secure communities. On a daily basis, individual police officers work to keep citizens safe and secure. Despite this, over recent years there have been numerous incidents of apparent police bias, which often seem to involve profiling based on demographics, and the use of excessive force (see Healy, 2014; Mackey, 2015; McKenna, 2015; Hasham, 2016).

These types of events involving what many believe to be biased and excessively violent behavior by individual police officers have led to public discourse in North America about police bias, and the related issue of the increasing militarization of police forces (Paul and Birzer, 2004). Given that many of the victims of alleged bias and brutality belong to visible minorities, questions of racism have also been raised. For example, in 2015 African Americans were shot dead by police officers at three times the rate of White Americans (Kindly, 2015). There is no doubt that the determinants of police behavior are complex and multi-faceted and these determinants presumably include factors such as police subculture, personality traits, increased media focus on violent crime, racial (and other demographic) stereotyping, and perhaps deficits in aspects of training related to situational assessments and decision making under stress (Herbert, 1998; Micucci and Gomme, 2005). Understanding how these factors interact to promote biased and sometimes aggressive behavior should be a key research goal for behavioral scientists. Indeed, if the complex interplay of factors that contribute to such thought and behavior can be understood, this knowledge could be leveraged to optimize police training, both with respect to cognitive skills and actual behavior.

One very visible component of police identity is the police uniform. Previous work has shown that police uniforms can induce feelings of safety in those around the uniformed person (Balkin and Houlden, 1983). Other research shows that uniforms are associated with the perception of increased competence, reliability, intelligence, helpfulness, status, and authority (Mauro, 1984; Singer and Singer, 1985; Lawrence and Watson, 1991; Durkin and Jeffery, 2000). Some early work showed that authoritarian uniforms are associated with perceptions of social power and increased levels of compliance from members of the public (Bickman, 1974). More recent work from sociology has also highlighted the symbolic link between modern day police uniforms and concepts of power and social control (Paul and Birzer, 2004). In fact, this work suggests an interesting effect of militarized uniform style on police culture. That is, not only is police culture symbolized in the uniforms worn by officers, the uniform, by virtue of its (paramilitary) style, also influences police culture. The notion that clothing conveys meaning is further illustrated by findings that clothing can have a more powerful role in impression formation than physical attractiveness (Conner et al., 1975). Much of the previous research on clothing (including uniforms) has tended to assess other peoples’ perceptions of the wearer. There are, however, a few exceptions to this, such as the famous Stanford Prison experiments, which showed that dressing like a guard or a prisoner had strong effects on subsequent (role specific) behavior (on student samples). These experiments explicitly used clothing to influence the thought and behavior of the wearer. Interestingly, since those experiments, though, there has been a little systematic attempt to understand how uniforms modulate thought and behavior in the wearer (Haney et al., 1973; Zimbardo, 2004).

Given the symbolic nature of clothing, an important question is whether the simple act of wearing a police uniform might affect cognitive processing in the wearer. Although the effects of clothing on cognition are understudied, some previous research has suggested that wearing certain clothing affects cognitive processing. In a key study conducted on university students, participants wearing a white lab coat showed improved performance on an attention task when the coat was “pitched” as a doctor’s coat, but not when it was pitched as a painter’s coat (Adam and Galinsky, 2012). Critically, this effect did not emerge when participants simply looked at the doctor’s coat (described to them as a “doctor’s coat”) lying on the table in front of them. This finding demonstrates that clothing *can* influence cognitive performance to a greater extent than we might expect for basic priming (i.e., from simply being exposed to the clothing). That is, *wearing* the clothing seems to be a critical factor in producing the observed effects. The authors coined the term “enclothed cognition” to signify that clothes themselves can exert measurable effects on cognitive processing. They further suggested that clothing may exert its effects partly due to the symbolic meaning attached to the clothing in question. Building on this notion of enclothed cognition, the current study investigated whether wearing a police-style uniform influences social cognitive processes in the wearer of the uniform. We focus specifically on whether wearing a police-style uniform affects social attention. Before outlining our hypotheses, it is important to consider what symbolic meaning might be imbued in police style uniforms.

There is good reason to believe that, among other things, police uniforms symbolize authority and power. For example, in one study an experimenter dressed like a police officer, randomly approached pedestrians, and ordered them to pick up an item, give a dime to another person, or step back from a bus stop. As a control, the experimenter alternatively wore casual clothes or a milk delivery uniform. Only the police-style uniform led to higher rate of compliance from citizens (Bickman, 1974). There is a vast social psychological literature on the effects of power on cognitive processing, and to the extent that the wearer of a police uniform feels powerful (Haney et al., 1973; Zimbardo, 2004), there are specific predictions that could be made about how their cognitive processing might be affected when wearing the uniform. However, before outlining these predictions, it will be useful to consider how uniforms might convey cultural meaning associated with the police subculture.

It is well known that clothing can signal many aspects of a person’s social identity including, but not limited to, socio-economic status, gender, religion, and occupation. Given this, police uniforms may symbolize meaning associated with the social identity of being a police officer (Feinberg et al., 1992). It has been pointed out that organizational dress symbolizes concepts related to the organization’s culture (Rafaeli and Pratt, 1993). Police officers operate in an occupational environment in which they frequently perceive the potential for danger and threat, and in which they are uniquely positioned to exercise coercive power (Paoline, 2003). Also, it has been argued that this perception of danger unifies police officers (i.e., creates a strong in-group identity) and serves to create barriers that separate them from the perceived source of that danger, which is the public (who may be conceived as an out-group) (Kappeler et al., 1998). In this way, a key part of police culture may involve being suspicious of the public and consciously or unconsciously viewing them as potential threats (Brown, 1988). It is important to note that members of the public often receive information about police through mass media (Reiner, 2010). Fictional representations of policing through, for example, TV series and movies, often portray police culture. While the accuracy of these portrayals may be questionable, some police shows depict police deviance and misconduct (e.g., rule-bending) as an essential part of effective policing (Dirikx et al., 2012). In view of this, we conjectured that asking students to wear a police-style uniform might prompt them to embody the social identity of a police officer (including the components of police culture as represented through the media).

Returning to the link between police uniforms and power, it is important to consider the kinds of effects that social power has on cognitive processing. Research shows that high-power individuals over-rely on stereotypes compared to low-power individuals (Depret and Fiske, 1993; Fiske, 1993), that people primed to high-power mirror others less than individuals primed to low-power (Hogeveen et al., 2013), and that high-power is associated with reduced perspective taking (Galinsky et al., 2006) and increased objectification (Civile and Obhi, 2016; Civile et al., 2016).

The over-reliance of power holders on stereotypes, in particular, might be especially important in the context of policing. Police often operate in time-sensitive and potentially threatening situations in which the use of stereotypes might hasten judgments about a specific social group member, and whether they pose a threat or not. To the extent that the police uniform itself symbolizes power and high vigilance to threat, we could reasonably predict that, when wearing the uniform, specific forms of bias may emerge. That is, we could hypothesize a kind of uniform induced attentional bias – in which attention is biased toward those members of social groups most associated with threat (rightly or wrongly). Given common stereotypes as well as results from numerous implicit association tests (Schaller et al., 2003), in the US, African American individuals might receive biased attentional processing and suffer from rapid stereotyping. In one study, it was found that when an actor was depicted as African American, rather than White, both White and African American participants found the actor’s behavior meaner and more threatening (Sagar and Schofield, 1980). More recently, Payne (2001) showed that under rapid decision conditions, participants are more likely to falsely report seeing a gun rather than a harmless object when they are primed with an African-American face, as opposed to a White face.

Furthermore, Correll et al. (2002) investigated the effect of race on a shoot/don’t shoot task comparing students with individuals in the community (adults recruited from various public places like bus stations and malls). Through the use of a simple videogame, participants were instructed to “shoot” armed targets and to “not shoot” unarmed targets. White university students (Correll et al., 2002, Experiment 1) made the correct response of quickly shooting when the target was African American more so than when the target was White. Interestingly, in a comparison study using a community sample (Correll et al., 2002, Experiment 4) both White and African American participants showed the same bias as that recorded in White students. Plant and Peruche (2005) extended this investigation to a sample of police officers with more than 2 years experience in law enforcement. Results showed that upon initial exposure to the computer simulation (shoot/don’t shoot Black and White suspects), police officers too were more likely to mistakenly shoot an unarmed Black suspect than a White one. However, after extensive training with the program the officers were able to eliminate this bias. Taken together these studies suggest that a racial bias, indexed as attentional response to certain stimuli, can be recorded in students as well as in adults in the community, and police officers.

In the Canadian context, such a racial bias might also emerge. However, there are important differences between the histories of many African Americans and African Canadians. Whereas the majority of African Americans may trace their origins in the US back to slavery, many African Canadians can trace their roots to voluntary immigration (Pabst, 2006). Thus, given this very different history, a racial bias for images of black faces^1^ vs. white faces for students in a Canadian sample, may not be as strong as that predicted for the US. In contrast, it could be expected that individuals from lower socioeconomic status (SES) backgrounds might capture the attention of individuals wearing a police uniform to a greater degree than individuals from higher socioeconomic groups.

To begin to examine these possibilities, we conducted three experiments examining how wearing a police-style uniform affects attention to social targets. In Experiment 1, we used a Drawn Attention task in which participants performed a shape categorization task in the presence of distractors that could either be white male faces, black male faces, individuals dressed in business suits or individuals dressed in hoodies. We chose business suits and hoodies as our manipulation of the SES of targets because these clothing types are thought to have strong pre-existing associations. For example, a previous study (McDermott and Pettijohn, 2011) showed that university students associated female models wearing hoodies with low SES when the logo on the hoodie was not from a prestigious brand. Also, the hoodie has become an index of a young person from the inner city, and a symbol of urban youth, who are commonly associated with violence and crime (Bell, 2013). A published report (Bawdon, 2009) about the media coverage and stereotypes linked to teenage boys, showed that the word “hoodie” often carries negative connotations. This leads to the potential creation of stereotypes that teenage boys who wear hoodies have criminal intent and are anti-social (see also Gatersleben et al., 2013 for a study that use “hoodies” to investigate social perception). Thus the hoodie has both social class and crime related associations. Therefore, in the experiments reported here we assume an association between low SES and images of people wearing a hoodies. In contrast, it can be argued that business suits are symbols of high-SES, and although they may have some associations to “white collar” crime, they are not as associated with violent crime as hoodies (Kraus and Mendes, 2014). Thus, we assume an association between high SES and images of people wearing business suits.

A primary function of visual-spatial attention is to enable rapid detection and analysis of new objects appearing in the environment (Yantis, 1996; Fox et al., 2000). Potentially, stimuli that are perceived as negative (e.g., a threat) are particularly important contenders for capture by the visual-attention system. Evidence for the propensity of negative stimuli to attract attention comes from research using tasks in which negative and neutral stimuli are placed in competition with each other and in which participants take longer to name the color of the negative stimulus compared to the neutral one. Thus, negative information captured attention of the participants leading to more interference on naming the color of the negative stimulus (Pratto and John, 1991; Le Doux, 1996). Similar tasks have been adopted in clinical studies (see Williams et al., 1996 for a review) and also in evolutionary psychology studies that investigated attentional disengagement from pictures of attractive men and women (e.g., Maner et al., 2007). Many researchers have suggested that these results reflect automatic drawing of attention toward negative stimuli or attractive stimuli in the context of a mating goal (see Maner et al., 2007). In line with this literature, in our Experiment 1 we asked participants to categorize images of shapes (targets) presented simultaneously with images of a distractor (black face, white face, hoodie, and suit). This task assesses the attentional salience of distractors indexed by performance when categorizing the shapes. We specifically predicted that when wearing a police-style uniform, individuals would be most distracted by black faces, and by individuals wearing hoodies (whom we propose will be associated with low SES). The response latency between the presentation of the target shape and the participant’s response constituted the reaction time (RT) measurement. Larger RTs indicated that the participant’s attention to the main task (i.e., categorization of the shape) was drawn away by the distractor stimulus. In Experiment 2, we aimed to directly assess whether visual attention is allocated toward the images of hoodies vs. suits, and black faces vs. white faces. In Experiment 2, we used a Dot-Probe task to assess the speed of responding to a target that appeared in a spatially congruent or incongruent location to a particular stimulus, drawn from the same set of distractor stimuli used in Experiment 1. Hence, attentional capture is measured by the RT to detect a small dot that appears at the bottom of one of our set of stimuli (black face, white face, hoodie, and suit). In the literature studies have found participants are faster to detect the dot when presented in a location congruent with negative (e.g., threat) stimuli (e.g., MacLeod and Mathews, 1988). The results from these two experimental paradigms should reveal whether attention in participants wearing the police-style uniform is biased toward specific types of social stimuli. In a control condition, we assessed attentional bias in participants wearing mechanic overalls, and participants not wearing any type of uniform. Finally, and most important, in Experiment 3 we aimed to replicate the results from Experiments 1 and 2 with a larger sample and we addressed the question of whether the effects of police-style uniforms are linked specifically to *wearing the uniform*, as opposed to just being *exposed to the uniform*.

## Experiment 1: Drawn Attention Task

### Methods

#### Participants

We recruited 14 students per sample group (*n* = 28, 5 male, 23 female; 16 Caucasian, 8 East Asian, 4 South Asian) from a Canadian university (mean age 18.67, range 18–22) who took part in the study for monetary compensation (10$ CAD). This experiment was approved by the institutional ethics review board. To calculate the sample size we used G^∗^Power software (Faul et al., 2007), assuming medium effect size *f* = 0.25, with two groups, and four within and between group measurements, which suggested a total sample of 28 participants in order to reach a statistical power of 0.81 (Cohen, 1988). Participants were asked to wear dark clothing and were randomly assigned to one of the two between-subjects conditions: police uniform or mechanic uniform.

#### Stimuli

In the Drawn Attention Task, we used a set of 64 faces (32 white and 32 black) selected from the previously validated 10k US Adult Faces database. These images have a resolution of at least 72 pixels/inch and have been cropped with an oval around the face to minimize background effects and resized to a height of 256 pixels with variable width (Bainbridge et al., 2013). For our experiment, these face images were edited. We whitened the backgrounds of each image around the face contours to eliminate the possibility that the color of the background could affect our results. All of the faces chosen for our experiment had a neutral expression with their eyes facing straight at the camera. Moreover, none of the faces chosen were showing teeth or had long hair obstructing the forehead.

Through an Internet search, we selected our set of SES stimuli. Specifically, we searched for males (standing in a neutral posture with arms down close to their body) wearing business suits to represent high-SES, and males wearing hoodies to represent low-SES. All images included the upper body, with faces cropped out, and part of the lower body of men wearing either a dark color business suit or a dark color hoodie and jeans or casual trousers. Just like for the face stimuli, we selected 64 (32 low-SES and 32 high-SES) SES stimuli. All the stimuli were standardized in a greyscale color on a white background using Gimp photo-editing software (Lecarme and Delvare, 2013).

We recruited three students and asked them to evaluate our SES images. Evaluators were instructed to rate the SES of the images they were about to see on the computer screen. A 5-point Likert scale was used with *1* indicating *Lower Class*, *3* indicating *Middle Class*, and *5* indicating *Upper Class*. All three evaluators rated images of suits higher than those of hoodies (*t*-tests comparing suits vs. hoodies showed a *p* < 0.001 in each evaluator). Evaluator n.1 (age 21, female) rated images of suits closer to the *Upper Class* (*M* = 4.25, *SD* = 0.77) and images of hoodies closer to *Lower Class* (*M* = 1.50, *SD* = 0.73). Evaluator n.2 (age 20, female) as well rated images of suits closer to the *Upper Class* (*M* = 4.06, *SD* = 0.77) and images of hoodies closer to *Lower Class* (*M* = 1.87, *SD* = 0.80). Similarly, evaluator n.3 (age 19, male) rated images of suits closer to the *Upper Class* (*M* = 4.43, *SD* = 0.62) and images of hoodies closer to *Lower Class* (*M* = 1.93, *SD* = 0.77).

#### Procedure

Participants were run in individual sessions. After signing the consent form, participants were given 10 min at most, alone in the testing room to put on either the police or mechanic uniform. In the testing room, there was a mirror that participants could use to ensure the uniform was put on properly.

**FIGURE 1:**
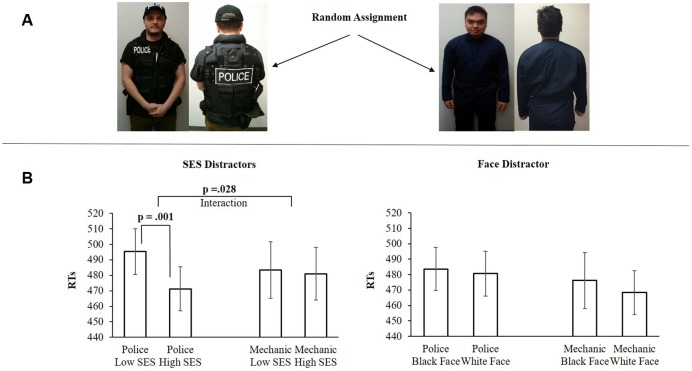
**(A)** Examples of the two sample groups. **(B)** The results from Experiment 1. The *X*-axis shows the stimulus conditions. The *Y*-axis shows the mean RTs (ms) for each condition. Error bars are SEM.

The mock police-style uniform included: Police t-shirt, police cap, police uniform belt, and police jacket. In the mechanic group, participants were asked to wear a mechanic’s coveralls (see **Figure 1A**). Once participants had indicated that they had put on the uniform, participants fetched the experimenter from the control room right next door to the testing room. The experimenter subsequently re-entered the room and provided instructions for the drawn attention task.

The Drawn Attention task was adapted from studies that have looked at face and body perception through the use of attentional distraction tasks (Lu and Chang, 2012) and attentional disengagement tasks (Maner et al., 2007). In the first trial, participants saw an instruction on the computer screen, informing them that, when the object appeared, their task was to categorize the object as a circle or a square by pressing either the “.” or “x” key on the keyboard. Participants were asked to respond as quickly and accurately as possible.

The procedure for each trial was as follows. First, a fixation cross (“+”) appeared in the center of the computer screen for 1000 ms. Next, a “target” object (either a circle or a square) was presented in one corner of the computer screen (i.e., upper left, upper right, lower left, and lower right). Simultaneously, a “distractor” stimulus (black face, white face, low-SES, or high-SES) appeared in either same location as the target (“filler trials”) or in the diagonally opposite corner (“drawn attention trials”). The target and distractor stimuli were displayed simultaneously for 800 ms.

The response latency between the appearance of the categorization object and the participant’s response constituted the RT measurement. Larger RTs indicated that the participant’s attention to the main task was drawn away by the distractor stimulus. Thus, drawn attention trials assessed the extent to which a particular stimulus type captured a participant’s attention.

The experiment consisted of 64 trials, consisting of 16 images from each distractor stimulus type: black face, white face, low-SES, or high-SES. Each stimulus was presented simultaneously with the circle or the square. Within each distractor stimulus type, eight stimuli were associated with the circle, and eight were associated with the square. The order of the trials was randomized and counterbalanced. Participants were not presented with the same distractor stimulus more than once, which was done in order to avoid any familiarity effects. At the end of the task, participants removed the uniform and were debriefed about the study.

#### Data Analysis

Our primary measure was RT responses. Each *p*-value reported in this paper is two-tailed, and we also report F or t value along with measures of variability (SE or SEM) and effect size ($\eta_{p}^{2}$). Since the aim of our study is to investigate two very different effects (racial bias and SES bias) we were not interested in directly comparing SES distractors and face distractors. Also, from the face perception literature, there have been several studies demonstrating that depending on the task used, faces and bodies may be perceived either similarly or differently (Slaughter et al., 2004). Thus, comparing directly faces vs. bodies risk to bring the discussion onto a completely different aspect of the study more related to the perceptual processes associated with the sets of stimuli. For these reasons, we conducted separate statistical analyses (ANOVA) for these distractor types between and within sample groups (*Police uniform*, *mechanic uniform*). Follow up, paired *t*-tests analyses were conducted to compare performance for white vs. black faces, and low vs. high SES in both police and mechanic uniform groups. Fortunately, an ANOVA is not very sensitive to moderate deviations from normality; simulation studies, using a variety of non-normal distributions, have shown that the false positive rate is not affected very much by this violation of the assumption (Glass et al., 1972; Harwell et al., 1992). This is because when you take a large number of random samples from a population, the means of those samples are approximately normally distributed even when the population is not normal. The same data analysis was adopted in Experiments 2 and 3. Additionally, in Experiment 3 we computed the Bayes factors (Dienes, 2011) to investigate further the level of confidence for the effects found.

Finally, in the three experiments reported here we assessed whether accuracy performance corresponding to each stimulus’ condition was above chance. *T*-tests analyses were conducted comparing accuracy for each stimulus’ condition against mean 50% (chance level). These revealed that in all three experiments the accuracy corresponding to each stimulus’ condition was significantly above chance (*p* < 0.001).

### Results

The RTs from all participants, measured in milliseconds, served as the primary measure for our statistical analysis. Collapsing RTs across target shape categories produced separate indices of attentional capture for each of the four distractor stimulus types. Trials in which the shape was incorrectly categorized were excluded from the analysis. Outliers (greater than 2.5 SD’s above the mean, smaller than 2.5 SD’s below the mean) were excluded in each participant data set. Such outliers accounted for 1.60% of the data. The participant with the highest number of outliers had a total of 3 (4.68%) of his scores.

#### Response Accuracy

Response accuracy was significantly above chance for both sample groups. In the *Police uniform* group, the percentage accuracy scores were as follows: Low-SES, *M* = 88%, *SD* = 10.24; High-SES, *M* = 87%, *SD* = 8.49; Black face, *M* = 87%, *SD* = 9.64; White face, *M* = 87%, *SD* = 9.80. In the *mechanic uniform* group, the percentage accuracy scores were as follows: Low-SES, *M* = 83%, *SD* = 10.23; High-SES, *M* = 86%, *SD* = 8.55; Black face, *M* = 88%, *SD* = 9.32; White face, *M* = 83%, *SD* = 10.16. No significant differences were found among the conditions.

#### Main Analysis: Reaction Time on Correct Trials

##### SES distractors

A 2×2 mixed model ANOVA using the within-subjects factors *SES* (low, high) and the between subjects factor *uniform* (police, mechanic) revealed a significant two-way interaction *F*(1,26) = 5.338, *p* = 0.028, $\eta_{p}^{2}$ = 0.172. Simple effect analysis shows that in the *police uniform* sample group, low-SES stimuli (*M* = 495.35 ms, *SD* = 55.33) were more attention-grabbing than high-SES (*M* = 471.20 ms, *SD* = 53.16) stimuli, *t*(13) = 4.146, *p* = 0.001, $\eta_{p}^{2}$ = 0.569. In the mechanic sample group, no differences were found between low-SES (*M* = 483.46 ms, *SD* = 68.56) stimuli and high-SES (*M* = 480.98 ms, *SD* = 63.59) stimuli, *t*(13) = 0.339, *p* = 0.740.

##### Face distractors

A 2×2 mixed model ANOVA using the within subjects factors *faces* (black, white) and the between subjects factor *uniform* (police, mechanic) revealed no effect of interaction *F*(1,26) = 0.264, *p* = 0.612. Simple effect analysis shows that in the *police uniform* sample group, no differences were found between black (*M* = 483.62 ms, *SD* = 52.16) and white (*M* = 480.66 ms, *SD* = 54.36) faces, *t*(13) = 0.463, *p* = 0.650. As well in the *mechanic* sample group, no differences were found between black (*M* = 476.14 ms, *SD* = 667.66) and white (*M* = 468.35 ms, *SD* = 53.62) faces, *t*(13) = 1.125, *p* = 0.280 (see **Figure 1B**).

##### Additional analyses between no uniform control and mechanic uniform

We ran a separate control group (*n* = 14; 14 female; 6 Caucasian, 5 East Asian, 3 South Asian; mean age 18.78, range 18–22) of participants who did not wear any uniform. As for the other two sample groups, participants in the *no uniform* control group were asked to wear dark color clothing the day of the experiment. After signing the consent form participants performed the computer task directly. The aim of running this supplemental group was to identify any potential bias induced by the mechanic uniform. The mean RTs for each stimulus condition were as follows: Low-SES, *M* = 460.40 ms, *SD* = 40.46; High-SES, *M* = 463.74 ms, *SD* = 49.63; Black face, *M* = 458.67 ms, *SD* = 46.54; White face, *M* = 467.08 ms, *SD* = 54.25. We ran two 2×2 mixed ANOVAs as in the main analysis reported above (i.e., separate ANOVA for each distractor type) and found no significant differences between the mechanic and the non-uniform groups (all tests, *p* > 0.5).

### Discussion

The results from Experiment 1 provide the first evidence that wearing a police-style uniform biases attention toward low-SES stimuli. Specifically, participants were slower in categorizing the target object (i.e., circle or square) when a low-SES image was presented simultaneously in a corner of the screen than when a high-SES image was presented in a corner of the screen. Crucially, this effect was not found for participants in the mechanic uniform group. We did not find any effects of the face stimuli in either the police or mechanic uniform groups. To further examine and corroborate this effect, in Experiment 2 we adopted a modified Dot-Probe task (MacLeod et al., 1986).

## Experiment 2: Modified Dot-Probe Task

### Method

The current task allows for studying the effects of uniforms on participants’ performance at detecting a dot probe (a black circle) presented simultaneously below one of two concurrently presented visual images. Visual images were selected from a stimulus set including black faces, white faces, low-SES targets, and high-SES targets (Staugaard, 2009). In this task, faster RTs to the dot indicate greater attention capture by the spatially aligned image.

#### Participants

**FIGURE 2:**
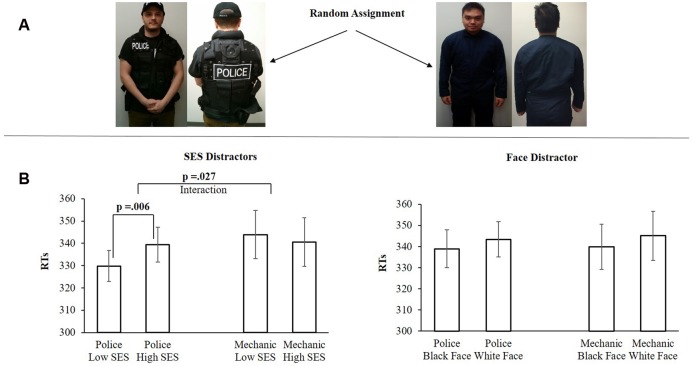
**(A)** Examples of the two sample groups. **(B)** The results from Experiment 2. The *X*-axis shows the stimulus conditions. The *Y*-axis shows the mean RTs (ms) for each condition. Error bars are SEM.

Fourteen new participants (also students who participated for monetary compensation) were recruited for each sample group in experiment 2 (*n* = 28, 6 male, 22 female; 9 South Asian, 7 East Asian, 6 Caucasian, 3 Middle Eastern, 2 African; mean age 19.5, range 18–24). As in Experiment 1, participants were randomly assigned to one of the two between-subjects conditions: Police uniform, mechanic uniform (see **Figure 2A**).

#### Stimuli

Experiment 2 adopted the same stimuli used in Experiment 1.

#### Procedure

The experimental procedure was similar to that used in Experiment 1. However, this time, we used a Dot-Probe task (Fashler and Katz, 2014). In the first event, participants saw instructions appear on the computer screen. Participants were instructed to respond when they detect a circle (i.e., probe) by pressing either the “.” when the circle appeared on the right side of the screen or “x” when the circle appeared on the left side of the screen. Participants were asked to respond as quickly and accurately as possible.

The procedure for each trial was as follows. First, a fixation cross (“+”) appeared in the center of the computer screen for 1000 ms. Next, a pair of faces (one white and one black) or SES stimuli (one low and one high) was presented in the center of the screen for 600 ms. In between each pair of stimuli (either the faces or the SES stimuli) a fixation cross was presented constantly to encourage fixation to the center of the screen. Simultaneously, a “probe” (i.e., black circle) appeared either below the stimulus (face or SES) on the right side or the one on the left side of the screen. The location of the dot was counterbalanced across the right and left side and for each stimulus’ types.

The response latency between the appearance of the dot and the participant’s response provided the RT measure. Shorter RTs indicated that attention was captured by the stimulus on the same side of the screen as the dot (by the white or black face, or toward the low or high-SES stimuli).

The experiment consisted of 64 trials, consisting of 16 images from each stimulus type: black face, white face, low-SES, or high-SES. Each stimulus was presented simultaneously with the probe either on the right side or the left side of the screen in a counterbalanced manner (eight to the right and eight to the left side). The order of all the trials was randomized and counterbalanced. Participants were not presented with the same stimulus more than once, in order to avoid any familiarity effects.

### Results

The RTs from all participants, measured in milliseconds, captured the speed of detecting the dot-probe. Collapsing RTs across left and right sides location of the probe produced indices of attentional capture for each of the four stimulus types. Trials in which the dot-probe was incorrectly detected were excluded from the analysis. Outliers (greater than 2.5 SD’s above the mean, smaller than 2.5 SD’s below the mean) were excluded in each participant data set. Such outliers accounted for 2% of the data. The participant with the highest number of outliers had a total of 3 (4.68%) of his scores.

Response accuracy was significantly above chance for both sample groups. In the *police uniform* group the percentage accuracy scores were as follows: Low-SES, *M* = 97%, *SD* = 4.03; High-SES, *M* = 96%, *SD* = 4.65; Black face, *M* = 92%, *SD* = 8.55; White face, *M* = 92%, *SD* = 6.56. No significant differences were found among the conditions. In the *mechanic uniform* group the percentage accuracy scores were as follows: Low-SES, *M* = 95%, *SD* = 6.68; High-SES, *M* = 97%, *SD* = 4.06; Black face, *M* = 97%, *SD* = 4.03; White face, *M* = 94%, *SD* = 6.23. No significant differences in accuracy were found among the conditions.

As for the Drawn Attention task in Experiment 1, in Experiment 2 we computed separate statistical analyses for the *SES* stimuli and the *Faces* stimuli between and within sample groups (*police uniform*, *mechanic uniform*).

#### Attentional Capture by SES Stimuli

A 2×2 mixed model ANOVA using the within-subjects factors *SES* (low, high) and the between subjects factor *uniform* (police, mechanic) revealed a significant two-way interaction *F*(1,26) = 5.456, *p* = 0.027, $\eta_{p}^{2}$ = 0.173. Simple effect analysis shows that in the *police uniform* sample group, the dot-probe was easier to detect when presented below the low-SES stimuli (*M* = 329.80 ms, *SD* = 25.97) than when it was presented below the high-SES (*M* = 339.40 ms, *SD* = 29.02) stimuli, *t*(13) = 3.275, *p* = 0.006, $\eta_{p}^{2}$ = 0.452. In the mechanic sample group, no differences were found between low-SES (*M* = 343.93 ms, *SD* = 40.64) stimuli and high-SES (*M* = 340.60 ms, *SD* = 40.74) stimuli, *t*(13) = 0.710, *p* = 0.490.

#### Attentional Capture by the Face Stimuli

A 2×2 mixed model ANOVA using the within subjects factors *faces* (black, white) and the between subjects factor *uniform* (police, mechanic) revealed no interaction effect *F*(1,26) = 0.011, *p* = 0.918. Simple effect analysis shows that in the *police uniform* sample group, no differences were found between black (*M* = 338.96 ms, *SD* = 33.42) and white (*M* = 343.45 ms, *SD* = 31.26) faces, *t*(13) = 1.150, *p* = 0.270. As well in the *mechanic* sample group, no differences were found between black (*M* = 339.93 ms, *SD* = 40.24) and white (*M* = 345.14 ms, *SD* = 43.32) faces, *t*(13) = 0.924, *p* = 0.372 (see **Figure 2B**).

#### Additional Analyses between No Uniform Control and Mechanic Uniform

As in Experiment 1, we ran a separate control group of participants (*n* = 14; 3 male, 11 female; 5 East Asian, 4 Caucasian, 3 South Asian, 2 African, mean age 19.78, range 18–27) who did not wear a uniform. After signing the consent form participants performed in the computer task. The mean RTs for each stimulus condition were as follow: Low-SES, *M* = 335.01 ms, *SD* = 34.91; High-SES, *M* = 334.17 ms, *SD* = 34.30; Black face, *M* = 338.16 ms, *SD* = 36.67; White face, *M* = 337.22 ms, *SD* = 37.26. We ran two 2×2 mixed ANOVAs as in the main analysis reported above (i.e., separate ANOVA for each distractor type) and found no significant differences between the mechanic and the non-uniform groups (all tests, *p* > 0.5).

### Discussion

In Experiment 2, we used a modified Dot-Probe task to further investigate whether wearing a police-style uniform induced an attentional bias to low SES stimuli. The results confirmed that, when a low and a high SES image are concurrently presented, participants are quicker to detect a Dot-Probe when it is spatially aligned with the low SES image. Interestingly, as in Experiment 1, there was no effect of the uniform for detecting the dot when it was spatially aligned with different race faces. This result bolsters the findings from Experiment 1 and constitutes strong evidence for police-style uniform induced biasing of social attention. One may argue that the effects we obtained in the police-style uniform sample (both in Experiments 1 and 2) may not be caused by wearing the uniform but perhaps just by being exposed to the uniform. Hence, participants may be primed to the symbolic meaning of being a police officer even just by seeing the uniform without having to wear it. Experiment 3 aims to address this issue by directly measuring the effects of *wearing* vs. *being exposed to* the police uniform on face and SES stimuli. Thus, we aimed to replicate the effects found in Experiment 1 and 2 (attentional bias toward low-SES vs. high-SES) for participants wearing the police-style uniform. In line with previous work on enclothed cognition (Adam and Galinsky, 2012), we predicted no attentional bias effects for participants who are simply exposed to the police-style uniform. Finally, we doubled the number of participants in each sample group (wearing the police uniform vs. being exposed to the police uniform) to further bolster our confidence in any observed effects.

## Experiment 3

### Participants

We recruited 28 students per sample group (*n* = 56, β = 0.99; 45 female, 9 male; 33 Caucasian, 13 East Asian, 6 South Asian, 3 Middle Eastern, 1 African) from a Canadian University (mean age 18.16, range 17–20) who took part in the study for credits. This experiment was approved by the institutional ethics review board. Participants were asked to wear dark clothing. Participants were randomly assigned to one of the two between-subjects conditions: Police uniform or control (police uniform located on the desk).

### Stimuli

Experiment 3 adopted the same stimuli as those used in Experiments 1 and 2.

### Procedure

Experiment 3 adopted a similar experimental procedure to that used in Experiments 1 and 2. However, Experiment 3 had participants in the *police uniform* group to wear the original campus police uniform from our University. This uniform was given to us by the University Security Office as a result of an ongoing collaboration. The uniform included: Police hat, police shirt (in different sizes), police protective vest, police pants (in different sizes), and police belt.

**FIGURE 3:**
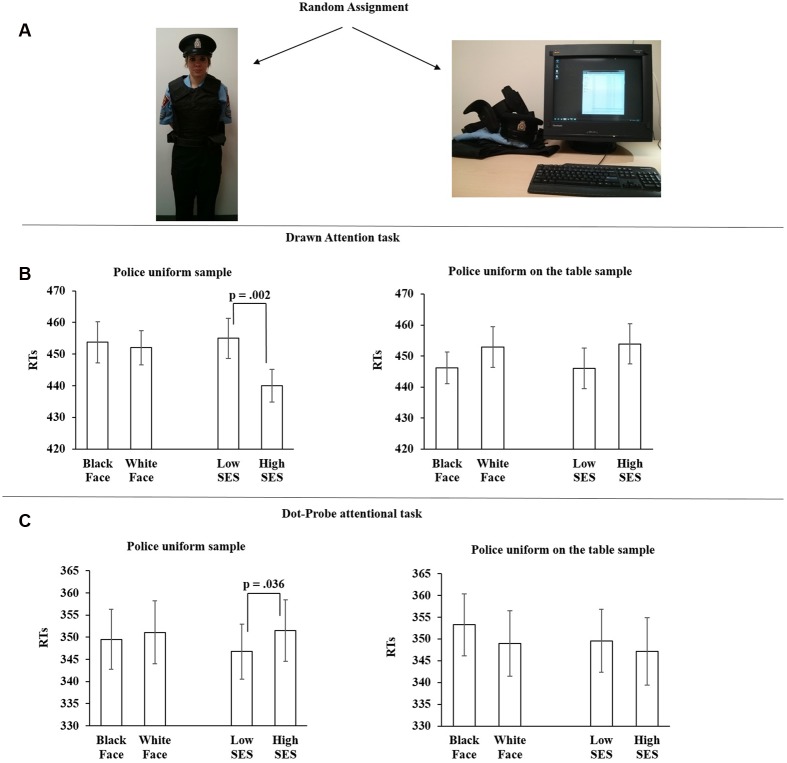
**(A)** Examples of the two sample groups. **(B)** The results from Experiment 3; Drawn Attention task. **(C)** The results from Experiment 3; Dot-Probe attentional task. For both **(B,C)**, the *X*-axis shows the four stimulus conditions. The *Y*-axis shows the mean RTs (ms) for each condition. Error bars are SEM.

In the *control group*, we tested whether the results obtained from the police uniform group were due specifically to wearing the uniform instead of simply being exposed to it. Thus, we positioned the police uniform on the desk next to the computer where participants performed the experiment (see **Figure 3A**). The uniform was located there before the participants entered the testing room and left there for entire the duration of the study. After signing the consent form participants performed the two computer tasks (Drawn Attention Task and Dot-Probe Task).

Each participant took part in both tasks (Drawn Attention Task and Dot-Probe Task), however, the order of presentation of the two tasks was counterbalanced across the participant sample. There was a short break of 1 min between the experiments to allow participants to rest their eyes. During this break, participants were not engaged in any conversation with the experimenter who was monitoring the study from the control room. Finally, the duration of stimulus presentation was standardized (600 ms) between the two attentional tasks.

### Results

#### Drawn Attention Task

As for Experiments 1 and 2, the RTs from all participants, measured in milliseconds, served as the primary measure for our statistical analysis. Collapsing RTs across target shape categories produced separate indices of attentional capture for each of the four distractor stimulus types. Trials in which the shape was incorrectly categorized were excluded from the analysis. Outliers (greater than 2.5 SD’s above the mean, smaller than 2.5 SD’s below the mean) were excluded in each participant data set. Such outliers accounted for 0.58% of the data. The participant with the highest number of outliers had a total of 3 (4.68%) of his scores.

##### Response accuracy

Response accuracy was significantly above chance for both sample groups. In the *police uniform* group, the percentage accuracy scores were as follows: Low-SES, *M* = 79%, *SD* = 10.24; High-SES, *M* = 81%, *SD* = 8.49; Black face, *M* = 85%, *SD* = 9.64; White face, *M* = 81%, *SD* = 9.80. In the *control* group the percentage accuracy scores were as follows: Low-SES, *M* = 82%, *SD* = 10.23; High-SES, *M* = 83%, *SD* = 8.55; Black face, *M* = 82%, *SD* = 9.32; White face, *M* = 82%, *SD* = 10.16. No significant differences were found among the conditions.

##### Main analysis: reaction time on correct trials

###### SES distractors

A 2×2 mixed model ANOVA using the within-subjects factors *SES* (low, high) and the between subjects factor *uniform* (police, control) revealed a significant two-way interaction *F*(1,54) = 9.339, *p* = 0.003, $\eta_{p}^{2}$ = 0.147. Simple effect analysis shows that in the *police uniform* sample group, low-SES stimuli (*M* = 454.99 ms, *SD* = 33.28) were more attention-grabbing than high-SES (*M* = 440.02 ms, *SD* = 27.52) stimuli, *t*(27) = 3.336, *p* = 0.002, $\eta_{p}^{2}$ = 0.292. In the control group, no differences were found between low-SES (*M* = 445.98 ms, *SD* = 34.86) stimuli and high-SES (*M* = 453.87 ms, *SD* = 34.21) stimuli, *t*(27) = 1.317, *p* = 0.199.

###### Face distractors

A 2×2 mixed model ANOVA using the within subjects factors *faces* (black, white) and the between subjects factor *uniform* (police, control) revealed no effect of interaction *F*(1,54) = 1.533, *p* = 0.221. Thus, simple effect analysis shows that in the *police uniform* sample group, no differences were found between black (*M* = 453.75 ms, *SD* = 34.35) and white (*M* = 452.01 ms, *SD* = 28.59) faces, *t*(27) = 0.356, *p* = 0.724. As well in the *control* sample group, no differences were found between black (*M* = 446.20 ms, *SD* = 20.80) and white (*M* = 452.90 ms, *SD* = 34.68) faces, *t*(27) = 1.410, *p* = 0.170 (see **Figure 3B**).

#### Bayes Factor Analyses for Low vs. High SES Stimuli

From both Drawn Attention tasks in Experiment 1 and Experiment 3, we have some evidence enabling us to claim that participants in the police uniform group show an attentional bias toward low-SES stimuli. As an additional analysis, we then calculated the Bayes factor using the procedures outlined by Dienes (2011). This used the effect in Experiment 1 as the prior, setting the standard deviation of *p* (population value | theory) to the mean for the difference between low and high-SES stimuli. We then used the standard error and the mean difference for low vs. high-SES effect found in Experiment 3. Finally, we calculated the Bayes factor assuming a one-tailed distribution for our theory and a mean of 0. This gave a Bayes factor (*B*) of 39.79 for the low vs. high-SES attentional bias effect in Experiment 3. This factor greatly exceeded 10 (Bayes factors more than 10 are considered decisive), providing a great deal of confidence in this finding (to see the Bayes factor calculator we adopted see Dienes, 2011).

#### Dot-Probe Task

As well as in Experiment 2, the RTs from all participants, measured in milliseconds, captured the speed of detecting the dot-probe. Collapsing RTs across left and right sides location of the probe produced indices of attentional capture for each of the four stimulus types. Trials in which the dot-probe was incorrectly detected were excluded from the analysis. Outliers (greater than 2.5 SD’s above the mean, smaller than 2.5 SD’s below the mean) were excluded in each participant data set. Such outliers accounted for 2.03% of the data. The participant with the highest number of outliers had a total of 3 (4.68%) of his scores.

Response accuracy was significantly above chance for both sample groups. In the *police uniform* group, the percentage accuracy scores were as follows: Low-SES, *M* = 93%, *SD* = 7.20; High-SES, *M* = 96%, *SD* = 5.67; Black face, *M* = 94%, *SD* = 4.06; White face, *M* = 95%, *SD* = 5.46. No significant differences were found among the conditions. In the *control* group, the percentage accuracy scores were as follows: Low-SES, *M* = 94%, *SD* = 7.08; High-SES, *M* = 96%, *SD* = 5.69; Black face, *M* = 93%, *SD* = 5.30; White face, *M* = 94%, *SD* = 6.99. No significant differences in accuracy were found among the conditions.

##### Attentional capture by SES Stimuli

A 2×2 mixed model ANOVA using the within-subjects factors *SES* (low, high) and the between subjects factor *uniform* (police, control) revealed a significant two-way interaction *F*(1,54) = 4.627, *p* = 0.036, $\eta_{p}^{2}$ = 0.079. Simple effect analysis shows that in the *police uniform* sample group, the dot-probe was easier to detect when presented below the low-SES stimuli (*M* = 346.74 ms, *SD* = 32.67) than when it was presented below the high-SES (*M* = 351.51 ms, *SD* = 36.54) stimuli, *t*(27) = 2.271, *p* = 0.006, $\eta_{p}^{2}$ = 0.452. In the control sample group, no differences were found between low-SES (*M* = 349.57 ms, *SD* = 38.21) stimuli and high-SES (*M* = 347.12 ms, *SD* = 40.92) stimuli, *t*(27) = 0.935, *p* = 0.358.

##### Attentional Capture by the Face Stimuli

A 2×2 mixed model ANOVA using the within subjects factors *faces* (black, white) and the between subjects factor *uniform* (police, control) revealed no interaction effect *F*(1,54) = 1.276, *p* = 0.264. Thus, simple effect analysis shows that in the *police uniform* sample group, no differences were found between black (*M* = 349.53 ms, *SD* = 35.58) and white (*M* = 351.09 ms, *SD* = 37.63) faces, *t*(27) = 0.430, *p* = 0.671. As well in the *control* sample group, no differences were found between black (*M* = 353.20 ms, *SD* = 37.60) and white (*M* = 348.95 ms, *SD* = 39.89) faces, *t*(27) = 1.157, *p* = 0.257 (see **Figure 3C**).

#### Bayes Factor Analyses for Low vs. High SES Stimuli

From both Dot-Probe tasks in Experiment 2 and Experiment 3, we have some evidence enabling us to claim that participants in the police uniform group show an attentional bias toward low-SES stimuli. As an additional analysis, we then calculated the Bayes factor using the procedures outlined by Dienes (2011). This used the effect in Experiment 2 as the prior, setting the standard deviation of *p* (population value | theory) to the mean for the difference between low and high-SES stimuli. We then used the standard error and the mean difference for low vs. high-SES effect found in Experiment 3. Finally, we calculated the Bayes factor assuming a one-tailed distribution for our theory and a mean of 0. This gave a Bayes factor (*B*) of 4.95 for the low vs. high-SES attentional bias effect in Experiment 3. This factor exceeded 3 (Bayes factors more than 3 are considered substantial), providing a great deal of confidence in this finding (for Bayes factor calculator see Dienes, 2011).

### Discussion

Experiment 3 is key to our investigation. Firstly, it replicated and extended our findings using a larger sample. Once again we found that wearing a police-style uniform elicited an attentional bias toward low-SES stimuli vs. high-SES stimuli. We now have three experiments showing this effect. Additionally, complementary Bayesian analyses using Experiment 1 (for the Drawn Attention task) and Experiment 2 (for the Dot-Probe task) to generate priors revealed that the Bayes factor for the effect of attentional bias for low-SES vs. high-SES stimuli was 39.79 in the Drawn Attention task and 4.95 in the Dot-Probe task. Thus, the overall Bayes factor obtained by multiplying the individual Bayes factor from each task (Drawn Attention and Dot-Probe task) comfortably exceeded 10 which suggested that we can be confident about our finding.

Importantly, Experiment 3 also showed that the results we obtained in the police uniform sample group hinge on the fact that participants are wearing the uniform, opposed to simply being exposed to it.

## General Discussion

In most developed countries, the police play an important role in enforcing the law, and protecting and serving the communities in which they operate. Despite being generally effective in their law enforcement duties, over recent years there have been numerous incidents in Canada and the US involving police aggression toward citizens. This has led to public discourse about why the police sometimes appear biased in their dealings with the public.

Whilst there is no doubt that numerous factors contribute to police thought and behavior, here we focused on the potentially biasing effects of police-style uniforms on attention to social targets. Across three experiments, when participants wore police-style uniforms, we found evidence for attentional bias toward social targets wearing hoodies (intended in our study to denote low SES). Specifically, in Experiment 1, participants wearing a police-style uniform were more distracted by images of individuals wearing hoodies, (indicative of low SES), compared to images of individuals wearing business suits (indicative of high SES). In Experiment 2, participants wearing police-style uniforms were quicker to detect a dot probe that was spatially aligned with images of individuals wearing hoodies compared to when the dot probe was spatially aligned with images of individuals wearing business suits. Importantly, these effects on attention were specific to wearing a police-style uniform and did not emerge in participants wearing mechanic overalls, or participants wearing their everyday clothes. Experiment 3 replicated these effects in a larger sample and crucially demonstrated that participants have to *wear* the police uniform for the effects to emerge. That is, simply being exposed to the uniform when it lay on the desk in front of them did not result in attentional bias to low SES stimuli. Our results suggest that the very act of putting on a police-style uniform introduces attentional bias toward a certain segment of the population. Furthermore, since what we attend to governs how we experience the world, uniform induced attentional biases have potentially far-reaching consequences. It is important to stress that, whilst we found reliable effects of police uniforms on attention, we did not assess the effects of uniforms on aggressive or violent behavior. Thus, whether our findings would translate into measurable effects on actual behavior remains an open question for future work.

One aspect of our results that was surprising was the lack of any effect for the face/race stimuli. More specifically, putting on the police-style uniform did not induce biased attention toward black faces compared to white faces. At first glance, this result is counter to what might be expected, given that many studies using implicit association (and other more explicit) measures have found strong associations between African Americans and crime (Payne, 2001; Greenwald et al., 2003; Eberhardt et al., 2004). Indeed, Eberhardt et al. (2004) reported that police officers are quicker to identify a dot when it was presented in the same location as a black face, compared to when it was presented in the same location as a white face. However, these authors did not specifically test whether this effect was in any way “uniform dependent.” Also, studies using the shoot/don’t shoot computer task paradigm showed that students, members of the public, and police officers all showed a bias toward African American targets compared to White targets. Importantly, these studies were conducted in the US, which has a sociocultural context quite different to that of Canada. Indeed, the lack of a face/race effect in our three Canadian samples of participants is noteworthy and highlights a potentially important difference between the Canadian and US cultural contexts. It is important to point out that Canada’s black population is much smaller than the black population in the US and that the latter has a “history, culture and level of social segregation different from that in Canada” (Manzo and Bailey, 2005). This specific sociocultural context in the US may explain why we did not find a race effect in our Canadian participants. This said it will be important for future studies to assess whether Canadian samples with different demographic characteristics, including rural/urban dwelling, for example, would show the same pattern of results we found here.

The fact that we found a reliable attentional biasing toward low-SES individuals is important, as it shows, for the first time, that the simple act of wearing a police-style uniform alters attentional systems, making the wearer hyper-vigilant to individuals who dress in a certain way. Given that the hoodie has strong associations to lower SES, this effect is tantamount to a kind of (unconscious) status profiling. Before dissecting our results in more detail, though, a word of caution, with respect to interpretation, is in order: Although, we intended the hoodie to symbolize lower socioeconomic class than the business suit, we must be careful in the specificity with which we interpret our results. That is, although hoodies are linked with low social class individuals, the media also creates associations between the hoodie and criminal activity, and race (McDermott and Pettijohn, 2008; Bawdon, 2009; Nguyen, 2015). So, while we provided evidence in support of the attentional bias effect we observed, we are not able to conclude whether the effect is due to a bias toward low social class targets or toward targets who are more associated with criminal activity. Whatever the exact association, when an observer puts on a police-style uniform, their attention *is* biased toward individuals wearing hoodies and this in itself has potential real-world consequences. Future work is needed to fully specify whether the hoodie is more or less associated with social class and/or crime *per se*.

Why might putting on a police-style uniform bias attention toward specific social targets? One potential explanation is that the police uniform symbolizes social identity – the identity of being a police officer – and the subculture of that group may be of critical importance when considering how uniforms might affect thought and behavior. Studies of police subculture have pointed out that the police frequently encounter dangerous and antagonistic individuals (Van Maanen, 1974; Cochran and Bromley, 2003) and therefore, an important part of police culture centers on a hyper-awareness of the threat, and a focus on mitigating this danger and threat. It has been suggested that the desire to mitigate danger and threat binds officers to one another, and unites them in creating a barrier between themselves and the perceived source of the threat – the public (Kappeler et al., 1998; Paoline, 2003). Given the fact that police subculture is portrayed in numerous movies and television shows, and sometimes in a negative way, police-style uniforms may automatically activate concepts associated with police culture even in individuals who themselves are not part of that culture (i.e., the students who participated in our study). Thus, putting on a police uniform may provide a top-down signal to the visual system to be hyper-sensitive to sources of danger and threat, in much the same way that providing top-down information about a target object feature can tune lower level sensory systems to that feature (Wolfe et al., 1989; Corbetta and Shulman, 2002).

Previous research has shown that police uniforms are associated with power and authority and thus, a person wearing a police-style uniform may feel powerful. Since power has been associated with an increased reliance on stereotypes, the combined effects of wearing a Police uniform may include threat vigilance and increased dependence on stereotyping. Thus, individuals wearing certain culturally meaningful clothing might become more salient to individuals wearing a police-style uniform. Our results are reminiscent of findings from cultural psychology studies showing that priming an interdependent or an independent self-construal (for example by using a pronoun circling task) changes attentional capture by flankers and performance on a local/global processing task (Lin and Han, 2009). Other brain imaging studies have found evidence that self-construal priming can affect brain activity at multiple levels in the information processing chain (Sui and Han, 2007; Lin et al., 2008). These results on self-construal priming would fall within the same category of “cultural priming” that we believe the current study focuses on. We suggest that, in our study, the combination of police uniforms symbolizing police subculture and inducing feelings of power creates a top-down attention biasing effect toward images of low SES individuals. However, these effects seem to be dependent on actually wearing the uniform, as they don’t emerge for individuals wearing mechanics overalls, their normal clothes or when the uniform is simply placed on the desk right in front of participants, for the duration of the experiment.

Another potential explanation is that the attentional bias effect toward low SES is caused by participant’s perceptions of police which they may begin to personally identify with when they put the uniform on. Given how much media attention “police bias” has received in the last few years, this may contribute to the development of attentional bias. However, considering several cases of police misconduct toward minorities reported by the media, it is surprising that participants only showed a bias toward low SES targets. Future studies should measure participant’s perceptions of police and police decision-making to determine whether wearing the police uniform induces participants to identify with positive or negative perceptions of the police and police culture.

We have discussed one limitation of our study above (i.e., the inability to be certain what social category the hoodie triggered in our participants’ minds), but there are others. Specifically, both experimental tasks employed in our study might be described as measuring attentional capture. That is, the degree to which attention is captured by a distractor (as in the drawn attention task) and the extent to which a particular image captures attention to a spatial location (thereby facilitating dot detection), do not tell us about whether attention would be maintained on the stimulus for further processing. Thus, although we can be quite sure that police-style uniforms result in attentional capture by images of people wearing hoodies, we cannot say anything beyond this. We cannot link our attentional effect to possible differences in sustained attention, or indeed to appropriate or inappropriate police behaviors. Future studies are needed to shed light on these possible consequences of police-style uniforms. In addition, future studies should explore the effects of uniforms on other social cognitive competencies such as perspective taking, empathy or theory of mind. Finally, future studies should investigate the idea that increased attentional focus to one type of stimulus might impair attentional processing of other stimuli (i.e., induce inattentional blindness; Mack and Rock, 1998; Simons and Chabris, 1999). Of course, this kind of effect in police work could have significant consequences where criminal acts may go unnoticed if an officer’s attention is directly elsewhere.

Our study is a confirmation and extension of the “enclothed cognition” effect reported by Adam and Galinsky (2012). Those authors found that wearing a white coat described as a doctor’s coat improved performance on a selective attention task and that simply being exposed to the coat did not produce the effect. Our results parallel this finding by showing that wearing a police uniform, but not simply being exposed to one, has measurable effects on social attention. We have suggested that uniforms may affect cognition by virtue of their cultural symbolization and that key features of police culture are power and threat vigilance. This research represents a step toward a more thorough investigation of how uniforms (and clothing more generally) affect a range of cognitive processes. If future work shows a link between uniform induced changes in attention and actual behavior, such work could be used to inform training programs for police officers in which the (unconscious) effects of wearing a uniform are highlighted, and strategies to mitigate any negative effects are suggested. In this regard, future work needs to determine what kinds of interventions might prevent the attentional biases that we have observed (and any related behavioral manifestations) from emerging. A key step in this process will be moving from student samples, to samples of real police officers, both when they are wearing a uniform and when they are not. Future work should also consider how different levels of the militarization of the uniform affect social cognition. That is, does increasing militarization of the uniform (e.g., by adding a weapon) exacerbate the effects we observed here?

In sum, across three experiments, we have demonstrated that wearing a police-style uniform biases attention toward individuals wearing hoodies. We have suggested that the power inducing effects of uniforms coupled with the associations of uniforms with increased threat vigilance (a critical aspect of police culture) might bias the attentional system toward stimuli that are perceived to represent danger and threat. While the current results do not speak directly to the kinds of aggressive incidents that have taken place over recent years in North America, this work is an initial step toward a broader understanding of the psychological effects of uniforms *on the wearer*.

## Ethics Statement

This study was approved by the Ethics Committee of McMaster University. The experiments were conducted in accordance with the Declaration of Helsinki. And all participants gave written informed consent after detailed explanation of the experiments.

## Author Contributions

CC and SO developed the idea behind the study and created the study design. Testing and data collection was conducted by CC. CC performed the data analysis and interpretation under the supervision of SO. CC draft the manuscript and SO provided critical revisions. All authors approved the final version of the manuscript for submission.

## Conflict of Interest Statement

The authors declare that the research was conducted in the absence of any commercial or financial relationships that could be construed as a potential conflict of interest.

## Footnotes

1 In the literature the term “black” is often used interchangeably with the term “African American.” We will use the terms “black” and “white” to describe our sets of African American and European (or Caucasian) American face stimuli.
